# A feasibility study on AI-controlled closed-loop electrical stimulation implants

**DOI:** 10.1038/s41598-023-36384-x

**Published:** 2023-06-22

**Authors:** Steffen Eickhoff, Augusto Garcia-Agundez, Daniela Haidar, Bashar Zaidat, Michael Adjei-Mosi, Peter Li, Carsten Eickhoff

**Affiliations:** 1 School of Sport and Exercise Sciences, Liverpool John Moores University, Liverpool, UK; 2 Brown Center for Biomedical Informatics, Brown University, Providence, RI USA; 3 Institute for Applied Medical Informatics, University of Tübingen, Tübingen, Germany

**Keywords:** Biomedical engineering, Electrical and electronic engineering

## Abstract

Miniaturized electrical stimulation (ES) implants show great promise in practice, but their real-time control by means of biophysical mechanistic algorithms is not feasible due to computational complexity. Here, we study the feasibility of more computationally efficient machine learning methods to control ES implants. For this, we estimate the normalized twitch force of the stimulated extensor digitorum longus muscle on n = 11 Wistar rats with intra- and cross-subject calibration. After 2000 training stimulations, we reach a mean absolute error of 0.03 in an intra-subject setting and 0.2 in a cross-subject setting with a random forest regressor. To the best of our knowledge, this work is the first experiment showing the feasibility of AI to simulate complex ES mechanistic models. However, the results of cross-subject training motivate more research on error reduction methods for this setting.

## Main

Electrical stimulation (ES) is a collective term for neuromodulation techniques that use artificial electrical fields to alter the state of voltage gated ion channels in the cell membranes of excitable tissue to control the release of neurotransmitters. ES finds application in many medical devices and therapies including the cochlear implant, the cardiac pacemaker, spinal cord, and deep brain stimulation. Most currently deployed ES implants have large overall volumes greater than 6000 mm$^3$, need to be placed in anatomical pockets, and require electrode leads to interface with the neural target tissue^1^. Although those existing systems have demonstrated successful and reliable stimulation performance over several years of implantation^2–4^, the degree of invasiveness of placing those large devices as well as the risk of electrode lead breakage represent major challenges to ES implantation in practice. These challenges are addressed by an ongoing ambition to develop miniaturized stimulation implants, such as so-called *electroceuticals*^5,6^, that are envisioned to be of the size of *“a grain of sand”* and to be placed minimally invasively directly in contact with the target excitable structure. Recent research assesses the feasibility of such novel therapeutic agents to treat an array of impairments and diseases including type 2 diabetes^7,8^, renal fibrosis, and hypertension^9^. A crucial feature of these and other novel ES implants will be their ability to provide feedback, *e.g.*, through an electroneurogram (ENG), about the degree to which the stimulation pulses activate (or suppress) the target tissue. Such forms of closed-loop control will be of utmost importance to the success of many ES applications, *e.g.*, those that seek to stimulate the autonomous nervous system^10^ to modulate hormone levels, where no obvious external feedback can feasibly be provided in real time.

**Figure 1:**
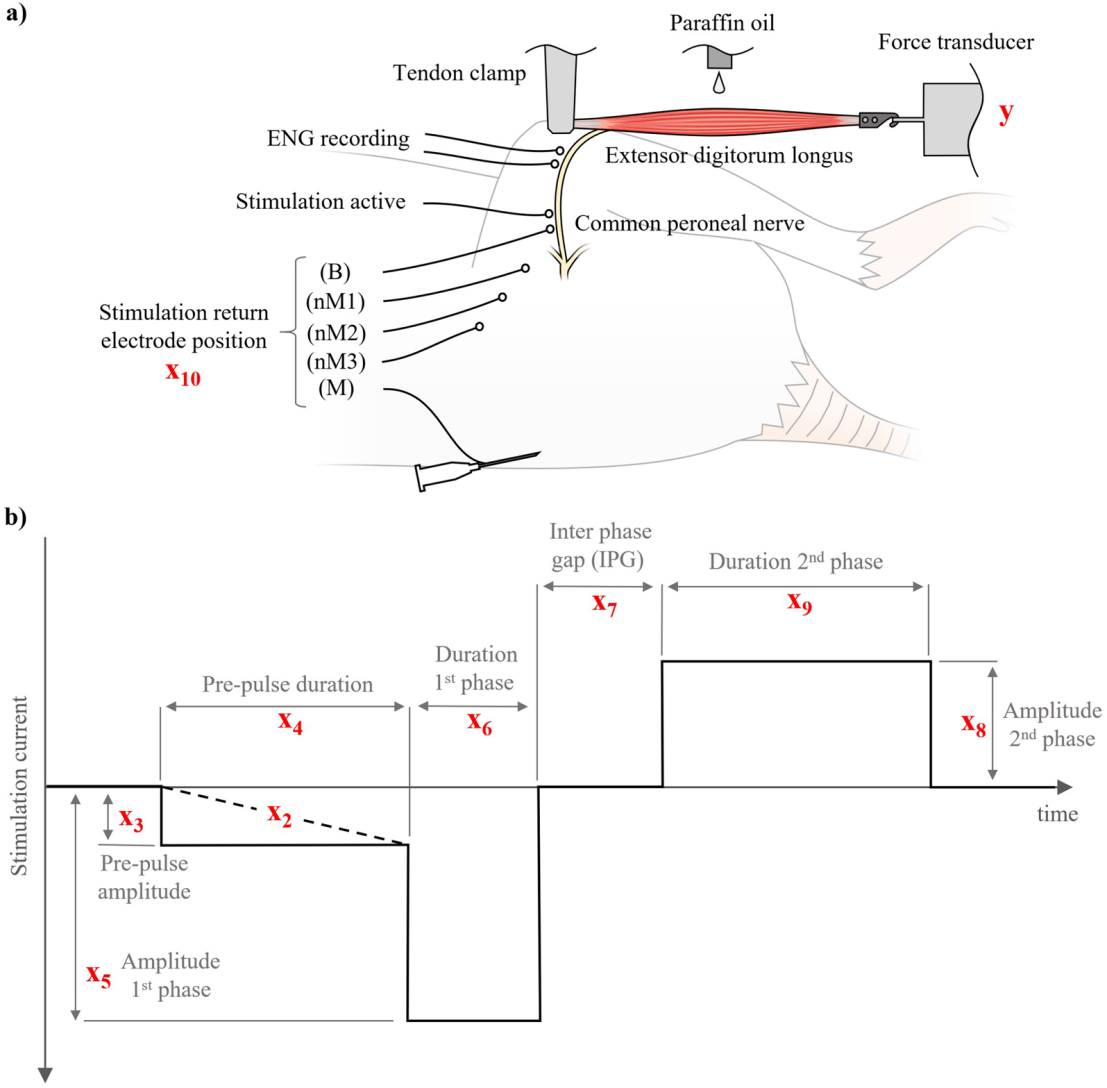
Experimental stimulation model and input/output variables. (**a**) Rodent in-vivo nerve-muscle preparation with different stimulation electrode configurations. The common peroneal nerve (CPN) in anaesthetized rats is stimulated and the isometric twitch force response of the extensor digitorum longus (EDL) muscle recorded. Depending on which return electrode is connected, the stimulation setup is either monopolar (with return M), bipolar (with return B), or “near monopolar” return electrode (return nM1-nM3). ENG recordings of the CPN were measured distally to stimulation. (**b**) Rectangular electrical stimulation pulses with and without pre-pulses and interphase gaps (IPGs) and with variable phase symmetry ratio are parameterized by variables x$_2$–x$_9$. Waveform not drawn to scale.

The input-output relationship of such closed-loop systems can be described and controlled by means of biophysical mechanistic models^11,12^. These methods, however, tend to be prohibitively computationally complex, preventing their real-time deployment and miniaturization. Computational statistical models such as developed via machine learning represent a promising alternative due to their significantly lower computational complexity and fast run-time performance^13^. The feasibility of artificial intelligence (AI) methods for predicting input-output functions for ES implants has been mentioned^14–16^, but many of these are theoretical works without experimental validation. The present study addresses this gap in scientific literature to prepare the way for AI-controlled closed-loop ES implants. We examined the performance of machine-learned regression models, such as multi-layer perceptron (MLP) and random forest (RF) regressors, to predict nerve activation with ES. These models were trained and tested on a dataset of terminal electrophysiological experiments in anaesthetized Wistar rats (Fig. 1a). Experiments included stimulation input parameters (such as amplitude, phase width, and interphase gap (IPG) duration) and the normalized neuromuscular output (Table 1) for 1926 electrical stimulations on average. We demonstrate a promising ES outcome prediction quality with mean absolute errors (MAE) of 0.03 for intrasubject calibration and 0.2 for cross-subject training.

## Results

**Figure 2:**
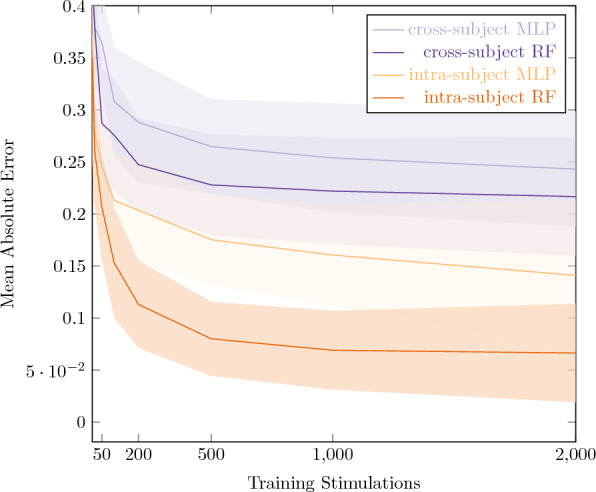
Electrical stimulation outcome prediction. Mean Absolute Error and 95% confidence interval for all experiments as a function of the number of training stimulations.

We studied the machine learning model under two experimental conditions for $n=11$ anaesthetized Wistar rats. Figure 2 displays the mean average error (MAE) and the 95% confidence interval for all experiments, while Table 2 presents the best achieved result for each experiment and its total run time.

### Intrasubject models

For this approach, a dedicated model was trained for each original subject. A small amount of the subject’s data served as training data to the models that were eventually evaluated on a held-out evaluation set from the same subject. The assignment between training and evaluation cohorts was made in non-overlapping random fashion. The random forest model seems to outperform and converge faster than the multilayer perceptron model, reaching a MAE of $0.03\pm 0.01$ shortly after 1000 training stimulations, as opposed to the multilayer perceptron, which converges at 2000 training stimulations with a MAE of $0.1\pm 0.02$. Furthermore, the total run time of the random forest was 2166 s, 45% that of the multilayer perceptron at 4864 s.

### Cross-subject models

The previously discussed experimental condition assumed that a small amount of data per subject could be obtained during a calibration phase before switching the model into prediction mode. In a second, more challenging, experimental condition, we assumed a case in which no such subject-specific calibration is possible. Instead, we trained a global model across $n-1$ subjects and used the remaining held-out subject for testing. We performed this evaluation *n* times, each time using a different subject for evaluation and report average performance across all subjects in Fig. 2. As expected, this setting cannot reach the same level of accuracy as intrasubject calibration. However, we observe result consistency: the random forest algorithm also outperforms and converges faster than the multilayer perceptron in this setting. It reaches a MAE of $0.2\pm 0.04$ converging after 500 stimulations, compared to the multilayer perceptron reaching a MAE of $0.22\pm 0.04$ requiring 2,000 stimulations to converge. The runtime comparison is also at 50%: 2910 s for the random forest compared to 5855 for the multilayer perceptron.

## Discussion

A discussion of the practical significance of the MAE reported here in predicting ES outcome is complicated by the fact that there is no established standard of minimum required accuracy for ES implant parameters. However, we choose the field of deep brain stimulation (DBS), that due to the complexity and density of excitable tissue of its stimulation target is likely to be among the first applications of miniaturized ES implants, as an exemplary reference. Studies that sought to investigate the relationship between DBS parameters and clinical response^17–19^ report relatively wide therapeutic windows for a variety of pulse widths, frequencies, and amplitudes. For stimuli with a pulse width of 60 μs, commonly suggested for DBS^20^, an effective therapeutic window of 2.2mA +/– 1.6mA has been reported for stimulation amplitudes in a range of 0–10 mA in four DBS implanted patients^19^. As this range of therapeutically effective amplitudes is significantly wider than the ES outcome prediction accuracy MAE of 0.03 reported here, the practical significance of our findings is supported.

**Figure 3:**
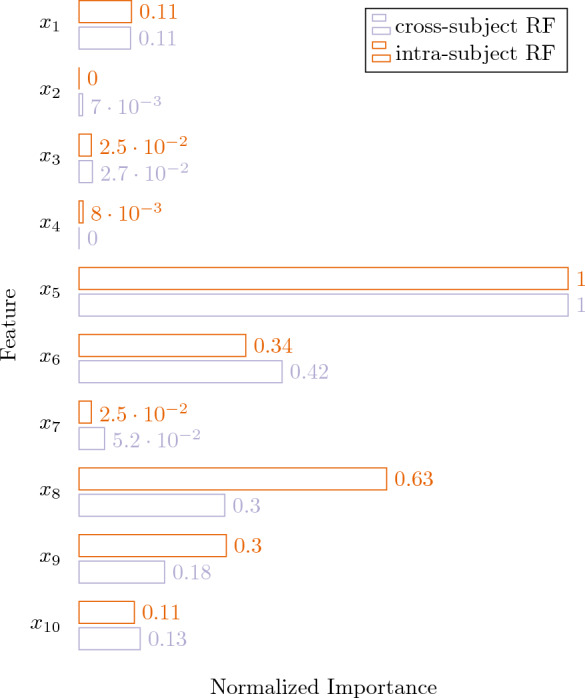
Normalized feature importance means for the random forest classifiers.

Overall, the practical feasibility of a 200-pulse calibration phase will strongly depend on the specific ES application. A key requirement is that the stimulation target must be eligible for intentional stimulation with 200 consecutive pulses. While this will be the case for many ES applications that target the central or peripheral nervous system, such as stimulation of the auditory nerve with cochlear implants, the same may not be feasible for ES devices that directly interfere with vital functions such as correction of critical heart failure by cardioverter defibrillators. Thus, for critical settings, more effective methods are required before translation to humans. Further, the practical feasibility will depend on the amount of time required to complete such a calibration phase and thus on the maximal stimulation pulse rate. Although the maximal feasible pulse rate depends on safety considerations^21,22^, the fatigue resistance of the stimulation target structure is more likely to be the limiting factor. Even with the low pulse rate of one stimulation every 3 seconds that we used in the terminal rat electrophysiology experiments to minimize neuromuscular fatigue^23,24^, a 200-pulse calibration phase would only require 10 minutes. As a measure of the models’ capability to transfer between the cross-subject and intrasubject settings, the average feature importances of the best-performing method, random forests, is provided in Fig. 3. We observe that both model families share identical rankings, mostly differing in the importance attributed to the second phase amplitude ($x_8$) and duration ($x_9$). In spite of this difference, and given the stochastic nature of these classifiers, we believe this is an indicator of the suitability of cross-subject approaches followed by a short intra-subject calibration period.

In this work, we observe a considerable difference in the performance of random forests and multilayer perceptrons. We hypothesize this is due to the fact that a random forest of sufficient depth can model richer interactions than a shallow multilayer perceptron. In addition, random forests are less data-hungry, and thus more efficient in resource-sparse environments. If associations are more complex, a random forest tends to capture them more effectively.

This study has a number of limitations. The normalization procedure of the output variable *y* (muscle twitch force response to electrical stimulation) represents a limitation of this study. The normalized twitch force response was capped at a value of 1, the amount of muscle tension that is produced when all motor neurons (and thus all motor units) are activated once. Some long duration pulses and those with long IPGs however, elicited significantly higher normalized muscle twitch force values. The rationale for this observation is that, when the electrical field transition within the stimulation pulse (compare Fig. 1b) are separated by a time greater than the absolute refractory period (ARP), some motor neurons can be activated a second time. This only happened at the upper range of tested stimulation phase widths (of approx. 800 μs and above) and only with the highest tested amplitudes. As this observation of multiple activations of motor neurons with single electrical pulses is subject of a separate, ongoing research study, we excluded this phenomenon by capping the output variable *y* at 1. For completeness, we also provide the experimental results without capping the output variable in the supplementary material in Table S1 and Figure S1.

A further element potentially limiting both benefit and applicability of AI methods for predicting ES input–output functions for closed-loop neuromodulation implants is the typically high degree of difficulty of measuring and normalizing neural activation. In our particular experimental setup we used normalized muscle twitch force as indication of neural activation, which has been verified to be positively correlated with the root mean square (RMS) of the electrically evoked compound action potential (eCAP) in the stimulated CPN^24^. However, systems that directly receive feedback from the stimulated nerve through an ENG, as would likely be necessary for miniaturized neurostimulation implants placed in direct proximity to the target nerve, will need to incorporate algorithms to analyse the ENG, increasing both computational complexity and overhead. A key challenge for such systems that receive feedback directly through ENG will be the distinction of eCAPs from stimulation artifact^25^ and/or other noise, such as strong bioelectric signal interference from muscle tissue. Biophysical mechanistic models are several orders of magnitude more computationally complex than the machine learned models presented here and are accurate to the point of not yielding any relevant predictive error. For this reason, a direct empirical comparison between our approach and a mechanistic model is omitted here.

The proposed random forest models used measured variables in temporal isolation. That means that, at each point in time, the model was provided with a range of input variables and was tasked with approximating the corresponding muscle twitch force at that time step. The model was oblivious of any previous or future steps in time. We chose this challenging setup as the most conservative estimate of machine learning system efficacy. It is plausible that future expansions to models that process the measured variables in their temporal progression (e.g., convolutional^26^, recurrent^27^ or transformer neural networks^28^) may reach even better approximation performance.

## Methods

### Surgical procedure and electrode placement

Experiments were conducted in 11 adult Wistar rats under adherence to the Animals (Scientific Procedures) Act of 1986 and approval by the Home Office under the project licence (PPL 40/3743) and are described in detail in^23,24^. This study was approved by the Liverpool John Moores University Research Ethics Committee.

Anaesthesia was introduced with isoflurane in oxygen at an initial concentration of 3%. A dose of 0.05 mg/kg of Buprenorphine (Temgesic, Indivior, Slough, UK) was administered intra-muscularly in the quadriceps muscle of the contralateral (right) hindlimb for analgesia. To maintain a stable deep level of anaesthesia throughout the duration of the experimental procedures, respiration rates were monitored and isoflurane concentrations readjusted accordingly between 1% and 2%. The anaesthetized animals were situated on an adjustable heat pad (E-Z Systems Corporation, Palmer, Pennsylvania, USA) to maintain the body core temperature between 37–38°C which was monitored using a rectal temperature probe.

For stimulation and electroneurogram (ENG) recordings, loop electrodes with an inner diameter of approximately 1mm were formed from the uninsulated ends of 40-strand stainless steel electrode wire (Electrode wire AS634, Cooner Sales Company, Chatsworth, California, USA). The loop electrodes were placed in the tissue immediately underneath the common peroneal nerve (CPN) and were slightly elongated to an oval form during placement and fixation. An injection of charge, the basis for any effect of ES, is only possible when both poles of the stimulator are connected via the tissue, *i.e.* when the electrical circuit is closed. The arrangement of these two poles of the stimulator, in relation to the excitable target structure, is referred to as *electrode configuration* or *mode*. In all experiments the active stimulation electrode was placed approximately 5mm distal from the point where the common peroneal nerve branches from the sciatic nerve. Thus, the position of the connected return electrode determined the stimulation electrode configuration. A second nerve electrode was placed 2mm distal to this stimulation electrode, and was used as return electrode in the bipolar stimulation configuration B. In some experiments, a second pair of electrodes, also with an inter-electrode separation of 2 mm, was placed approximately 10 mm distally to the stimulation electrodes to record a distal ENG of the stimulated nerve. After placing the nerve electrodes, the muscle and skin were closed to prevent the nerve from drying out. In experiments 3–5 three additional loop electrodes were fixed to the muscle tissue approximately 5, 10, and 15 mm away from the CPN and served as return electrode in the *“near monopolar”* configurations nM1 (5 mm), nM2 (10 mm), and nM3 (15 mm). Further, a hypodermic needle (21G x 1-1/2”) was placed in the dorsal skin of the animals and was used as return electrode for the monopolar configuration M. While the return electrode, and thus the stimulation electrode configuration, was selected and connected manually in experiments 1–6, return electrode connection was achieved automatically with a computer-controlled relay unit in experiments 7–11.

The extensor digitorum longus (EDL) muscle was accessed by dissection of the distal tibialis anterior (TA) tendon and careful blunt dissection the TA muscle belly from the deeper lying EDL. The proximal tendon of the EDL was freed and clamped with a sturdy artery forceps that was firmly mounted on the experimental steel table. The distal EDL tendon was cut and connected with a miniature hook to a force transducer (Gould Inc, Statham Instrument Division, Oxnard, California, USA) which was also fixed to the steel table. Thus, EDL innervation and blood supply were preserved and the muscle was mechanically isolated and held in an isometric condition. The muscle length was set by increasing it from slack in 0.5 mm increments to the ideal length, at which the developed isometric twitch force response to single electrical stimuli was maximal but without excessive passive muscle tension^29^. To maintain physiological muscle temperature and to prevent the muscle from drying, heated liquid paraffin oil was delivered by a peristaltic pump (Watson-Marlow Ltd., Falmouth, Cornwall, UK) at a rate of 0.1ml/min to the EDL muscle surface. Dehydration during the experimental procedures was prevented by subcutaneously administration of approximately 1ml of sterilized saline solution (OXOID Ltd., Basingstoke, Hampshire, UK) per hour.

### Stimulation equipment and protocols

Stimulation pulses were generated in LabVIEW$^{\text {TM}}$2016 (National Instruments Corporation, Austin, Texas, USA) and delivered at a resolution of 1 MS/s over the analog output of a NI PCIe 6351 Data Acquisition Card (National Instruments Corporation, Austin, Texas, USA) to a galvanically isolated voltage-to-current converter. The pulses were delivered to the active stimulation electrode at the CPN at a rate of one pulse every three seconds.

Pulses with varying combinations of the pulse parameters to be tested in each specific experiment (e.g. amplitude, phase width (phw), interphase gap (IPG) duration, and return electrode configuration) were delivered in random order. A control pulse for muscle response normalization was delivered after every 20 test stimulations. These control pulses were biphasic (cathodic phase first) rectangular pulses with phase width of 200 μs and amplitudes set for each experiment separately (typically 1 mA) to elicit maximal isometric twitch force. Twitch force responses to the test stimulations were divided by the closest control pulse and thus normalized. Randomization and normalization of test pulses allowed us to attain recordings that are not affected by progressive or cyclic variations in body temperature, depth of anaesthesia or fatigue.

While most test stimulations were variations of biphasic or monophasic rectangular pulses (*e.g.*, with pre-pulse or IPG), in experiments 1–6 a subset of Gaussian-shaped pulses was tested. The kurtosis of the current waveform of these pulses is described by:

1 $$\begin{aligned} I(t)=e^{-\frac{\left( \frac{t}{phw}-0.5\right) ^2}{0.045}}. \end{aligned}$$

### Recording equipment and settings

Isometric twitch force responses of the EDL muscle and the ENG of the stimulated CPN were recorded at a sample rate of 100 kS/s. The signals were recorded with a PowerLab 16/35 (ADInstruments Pty Ltd, Bella Vista, New South Wales, Australia) and stored, pre-processed and exported using LabChart 7 Pro (ADInstruments Pty Ltd, Bella Vista, New South Wales, Australia).

### Data analysis and statistical tests

The developed isometric muscle twitch force was determined by subtraction of the passive muscle tension (typically between 0.05 and 0.1 N) from the peak recorded muscle force value. Developed twitch force responses to test stimulations were normalized by division by the nearest control pulse as introduced above.

### Data structure

The data of each terminal rodent electrophysiology experiment is contained in a separate data set in which each individual electrical stimulation pulse is represented by a column (Table 1). The first input variable x$_0$ is the time at which the pulse is delivered, measured in seconds from the beginning of the experiment (after the surgical procedures, *i.e.*, the first stimulation is delivered at x$_0$ = 0). x$_1$ denotes the type of stimulation event, 0 indicating control pulses that were used for data normalization, a value of 1 stands for rectangular pulses, and 2 for Gaussian-shaped (Equation 1) stimuli. Pre-pulses were only used in experiments 7–1 and thus only with rectangular rather than Gaussian stimulation pulses. The pre-pulse shape is defined by variable x$_2$ (0 = no pre-pulse, 1 = ramped pre-pulse, 2 = square pre-pulse). Variable x$_3$–x$_9$ further parameterize shape and amplitude of the stimulation pulses as illustrated in (Fig. 1b). x$_{10}$ describes the return electrode position and thus the stimulation electrode configuration or mode: 1 = monopolar (M), 2 = bipolar (B), 3 = near-monopolar (nM1), 4 = near-monopolar (nM2), and 5 = near-monopolar (nM3). The output variable y is the normalized twitch force between 0 and 1.

### Machine learning

We trained random forest^30^ and multilayer perceptron models^31^ to infer normalized twitch force *y* based on the measured variables $x_0 \ldots x_{10}$ presented in Table 1. The model was implemented in Python using the Scikit-learn library version 0.22. Model hypeparameters were empirically determined via cross-validation on the training set as follows. For the random forest, intra-subject calibration: mean squared error loss, no feature subset selection, 5-fold cross validation, 1000 estimators, random seed 42. Cross-subject calibration: minimum 10 samples per split, 50 estimators, otherwise identical. For the multilayer perceptron, intra-subject calibration: tanh activation function, hidden layer size 40, max iterations 1000, Adam optimization, 5-fold cross validation, random seed 42. Cross-subject calibration: logistic activation function, hidden layer size 20, otherwise identical. Model training was performed on an Intel Core i7 8700K CPU @ 3.7 GHz, with the experiment run times described in Table 2. Approximate model execution time on single test sets for all four experiments are included in Table 2.

**Table 1:** Exemplary stimulation input/output data.

x$_{0}$	T (s)	.	4737	4740	4743	4746	4749	.
x$_{1}$	Event	.	0	1	1	1	1	.
x$_{2}$	Pre-pulse shape	.	0	1	1	1	1	.
x$_{3}$	Pre-pulse amplitude (μA)	.	0	175	110	92.5	170	.
x$_{4}$	Pre-pulse duration (μs)	.	0	5000	5000	10,000	10,000	.
x$_{5}$	Amplitude 1st phase (μA)	.	0	1750	1100	925	1700	.
x$_{6}$	Duration 1st phase (μs)	.	0	40	40	40	40	.
x$_{7}$	Interphase gap (μs)	.	0	0	0	0	0	.
x$_{8}$	Amplitude 2nd phase (μA)	.	0	-1750	-1100	-925	-1700	.
x$_{9}$	Duration 2nd phase (μs)	.	0	40	40	40	40	.
x$_{10}$	Return electrode position	.	0	2	1	1	1	.
y	Normalized twitch force	.	1	0.9853	0.0444	0.0007	1.0052	.

We trained and tested the machine learning model under two experimental conditions:

Intra-subject.: In this condition we trained a dedicated model for each original experiment. The available rows corresponding to non-control pulses are randomly assigned to the training (80%) and test sets (20%). Performance was measured on the subject-specific test set and reported in the form of averages across all subjects. The exact amount of available training data was iteratively increased between $k = 10$ and up to $k=2000$ rows of measurements. For each iteration, subjects with less measurements than *k* were excluded (see Table 3). To limit sampling-induced variance, each setting of *k* was sampled 20 times and the numbers reported in Fig. 2 represent averages across those 20 runs.
Cross-subject.: In this condition, we trained a single global model using all rows of $n-1$ subjects for model training and all rows of the remaining held-out subject for testing. We perform this evaluation *n* times, each time using a different subject for evaluation and report average performance across all subjects.

**Table 2:** Results of all four methods after 2000 training stimulations.

Method	Intrasubject MLP	Intrasubject RF
MAE Mean (%)	0.10	0.03
MAE Std (%)	0.02	0.01
Total Runtime (s)	4864	2166
Test Set Runtime Mean (s)	0.0014	0.0446
Test Set Runtime Std (s)	0.0006	0.0418

Method	Cross-Subject MLP	Cross-Subject RF
MAE Mean (%)	0.22	0.20
MAE Std (%)	0.04	0.04
Total Runtime (s)	5855	2910
Test Set Runtime Mean (s)	0.0054	0.0969
Test Set Runtime Std (s)	0.0030	0.1008

Mean and standard deviation are adimensional. Total runtime represents total experiment runtime per method.

### Performance metric

Model performance is measured in terms of mean absolute error (MAE). It is defined as the distance between the experimentally observed muscle twitch force *y* and predicted twitch force $y^*$ for each measurement, averaged across *n* measurements.

$$\begin{aligned} {MAE} = \frac{1}{n} \sum _{i=1}^n |y_i - y_i^*| \end{aligned}$$

This metric is agnostic of error direction (*i.e.,* over and under estimates of twitch force are conflated into the magnitude of the error but do not cancel one-another out). As our ground truth and predicted normalized twitch force are bounded in the [0, 1] interval, so are the corresponding errors.As a consequence, we can conveniently interpret MAE scores as absolute error percentages ranging from perfect predictions (MAE = 0) to maximally wrong predictions (MAE = 1).

### Ethics statement

This study is reported in accordance with the ARRIVE guidelines, and was approved by the Liverpool John Moores University Animal Welfare and Ethical Review Board.

**Table 3:** Length of input data for each subject.

Subject ID	Number of stimulations
JFTO 126	739
JFTO 127	442
JFTO 128	2493
JFTO 129	2216
JFTO 131	1108
JFTO 132	2216
JFTO 144	2040
JFTO 175	2492
JFTO 176	2480
JFTO 204	2480
JFTO 205	2480

## Supplementary Information

Supplementary Information.

## Data Availability

All experimental data used for this study can be obtained from https://data.mendeley.com/datasets/9pnx7nj327/1.

## Code availability

We share our code base in the form of an interactive Jupyter notebook that reproduces all numbers and figures presented in this manuscript. All program code is available from https://github.com/bcbi-edu/p_eickhoff_estim.
