# Supplementary material for: A feasibility study on AI-controlled closed-loop electrical stimulation implants
Source: Sci Rep. 2023 Jun 22;13:10163. doi: 10.1038/s41598-023-36384-x (PMC10287710; doi:10.1038/s41598-023-36384-x)
Supplement: Supplementary file 1 — Supplementary Information. [file 41598_2023_36384_MOESM1_ESM.pdf]

A Feasibility Study on AI-Controlled Closed-Loop Electrical Stimulation Implants

Steffen Eickhoff[1], Augusto Garcia-Agundez[2], Daniela Haidar[2], Bashar Zaidat[2], Michael Adjei-Mosi[2], Peter Li[2], and Carsten Eickhoff[2*]

[1] School of Sport and Exercise Sciences, Liverpool John Moores University

[2] Brown Center for Biomedical Informatics, Brown University, USA

∗ 233 Richmond St, 02903 Providence RI, USA

Supplementary Data 1: Additional experiments

In this section, we describe the experiment results without capping the output variable y value at 1. This results in a performance loss of 0.02 to 0.04 for all MAE values. Results are presented in Figure S1 and Table S1, and are directly comparable to the manuscript's Figure 2 and Table 2.

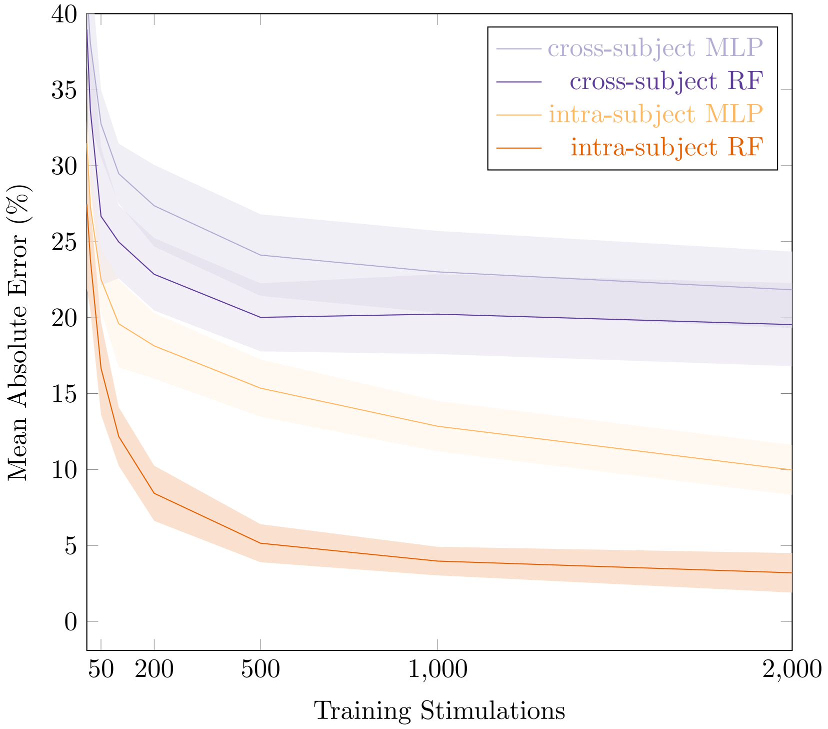

Figure S1: **Electrical stimulation outcome prediction with an uncapped output variable.** Mean Absolute Error and 95% confidence interval for all experiments as a function of the number of training stimulations

Table S1: **Results of all four methods after 2000 training stimulations with an uncapped output variable.** Mean and standard deviation are adimensional. Total runtime represents total experiment runtime per method

**Method**	**Intrasubject MLP**	**Intrasubject RF**
MAE Mean (%)	0.14	0.07
MAE Std (%)	0.08	0.05
Total Runtime (s)	3283	1827
Test Set Runtime Mean (s)	0.0006	0.0484
Test Set Runtime Std (s)	0.0005	0.0445
**Method**	**Cross-Subject MLP**	**Cross-Subject RF**
MAE Mean (%)	0.24	0.22
MAE Std (%)	0.08	0.08
Total Runtime (s)	4420	2550
Test Set Runtime Mean (s)	0.0021	0.1119
Test Set Runtime Std (s)	0.0008	0.0984
